# A Descriptive Investigation of Infant Feeding Bottles Marketed in the UK Designed to Replicate Breastfeeding and the Evidence That Underpins Them

**DOI:** 10.1111/mcn.70008

**Published:** 2025-03-02

**Authors:** Clare Maxwell, Becky Self, Kathryn Bould

**Affiliations:** ^1^ School of Public and Allied Health Liverpool John Moores University Liverpool UK; ^2^ School of Psychology Liverpool John Moores University Liverpool UK

**Keywords:** bottle feeding, breastfeeding, combination feeding, infant feeding, infant feeding bottles, JBI appraisal, marketing

## Abstract

Scant attention has been given to the marketing of infant feeding bottles and teats with claimed equivalence to breastfeeding. Such bottles are purported as having ‘breast‐like’ qualities and to be interchangeable with breastfeeding, encouraging breastfeeding mothers to combine breast and bottle feeding. However, the introduction of bottle feeding alongside breastfeeding can have a negative impact on breastfeeding duration and lead to cessation. We investigated features of infant feeding bottles marketed in the United Kingdom to replicate breastfeeding and appraised the underpinning evidence. We searched online to identify the most popular bottles marketed for breastfeeding in the United Kingdom and captured marketing materials from the bottle brand websites, importing them into NVivo11 for data analysis. We coded data in relation to features of bottles associated with breastfeeding and used Johanna Briggs Institute (JBI) critical appraisal tools to appraise the evidence used to underpin the bottle features. We identified 10 bottle brands and 8 main advertised features of bottles aligned to breastfeeding. Features included bottles that simulated the breast, imitated breastfeeding physiology and aided combined breast and bottle feeding. Scientific evidence to support the bottle features was scarce, misleading, and inadequate, with only one study deemed to be high quality. Our findings show that infant feeding bottles are being marketed as equivalent to breastfeeding; however, the scientific evidence used to support features of these bottles is almost non‐existent. Research on the impact of the marketing of bottles on breastfeeding and more effective controls of bottle company advertising are needed.

## Summary

- Infant feeding bottles and teats are being marketed as ‘equivalent’ to breastfeeding, with claims that they can replicate and in turn replace breastfeeding.
- Marketed features include bottles and teats that simulate the breast, imitate breastfeeding physiology and aid combined breast and bottle feeding.
- The scientific evidence used to underpin claims around the bottle and teat features is extremely limited, almost universally of low quality and at times misleading.

## Graphical abstract

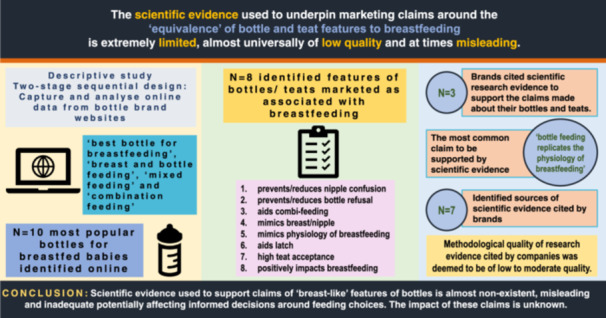

## 1 Introduction

Breastfeeding is clearly associated with short‐, medium‐ and long‐term benefits for mothers and infants (Victora et al. 2016) with the WHO recommending exclusive breastfeeding for 6 months and alongside complementary foods for up to 2 years or beyond (WHO 2023). However, less than 50% of babies worldwide are breastfed, with the socio‐cultural ‘norm’ being formula feeding via a bottle in an increasing number of countries (Rollins et al. 2023). For England, the rate of exclusive breastfeeding at 6 months is < 1% (McAndrew et al. 2012), one of the lowest in the world.

The latest Lancet 2023 Breastfeeding Series emphasises how exploitative marketing of formula milk has a catastrophic impact on breastfeeding, with implications that formula milk is equivalent to breastmilk (Rollins et al. 2023). Scant attention however has been given to the marketing of infant feeding bottles and teats (for the purpose of this paper we will refer to infant feeding bottles and teats as ‘bottles’) and their claimed equivalence to breastfeeding. Such bottles are marketed as having ‘breast‐like’ qualities and to be interchangeable with breastfeeding, promoting and encouraging breastfeeding mothers to combi‐feed^1^ or to ‘breastmilk feed’.^2^

Although is difficult to isolate the impact bottle feeding as a mode of infant feeding has on breastfeeding, there is evidence that combi‐feeding can impact duration of breastfeeding negatively both when infant formula (Michalopoulou et al. 2024; Pérez‐Escamilla et al. 2022) and expressed breastmilk (EBM) (McCoy and Heggie 2020; Forster et al. 2015) is given. In addition, bottle feeding containing formula or EBM can lead to childhood obesity due to lack of self‐regulation of intake by the infant and caregiver pressure (Ventura et al. 2020; Zheng et al. 2020). And although the evidence to support ‘nipple confusion’, defined by Neifert et al. (1995) as ‘a neonate's difficulty …to extract milk from the breast after exposure to an artificial teat’ continues to be inconclusive (Zimmerman and Thompson 2015), avoidance of bottles during the establishment of breastfeeding in preterm infants has been found to have a positive impact on breastfeeding (Allen et al. 2021). In essence, bottle feeding a breastfed baby has the potential to shorten breastfeeding duration and increase cessation.

The marketing of bottles is covered by the WHO International Code of Breastmilk Substitutes hereafter referred to as ‘the Code’ (WHO 1981). This requires advertising to refrain from idealising bottles or suggesting their equivalence to breastfeeding. Advertising should also include information on the benefits and superiority of breastfeeding. Although 146 WHO member states have adopted legal measures aligned to the Code provisions, only 33 are substantially aligned with the Code (WHO, UNICEF, IBFAN 2024). Legally, the United Kingdom is classified as aligning with ‘some provisions of the code’ with bottles and teats excluded from any legal measures. In England (and the rest of the United Kingdom) regulation of bottles does not refer to the WHO Code or the possible negative impact they can have on breastfeeding.

In England (and the rest of the United Kingdom) regulation of bottles is not explicit in terms of the context of breastfeeding. However, advertising can be considered to come under legislation on ‘Requirements on information relating to infant and young child feeding’ (Gov.UK 2016) which requires infant feeding information to state the negative effect on breastfeeding of introducing partial bottle feeding. This is also covered by the WHO code. However, regulation of bottles across the United Kingdom and application of the Code is limited, with the emphasis being on infant formula.

The marketing of bottles that are purported to exhibit ‘breast‐like’ features has the potential to encourage combi‐feeding and negatively impact existing poor breastfeeding rates. This in turn will deny the health benefits associated with breastfeeding to mothers and babies. To date, no studies have investigated the features of such bottles marketed in the United Kingdom or the evidence that the bottle companies use to underpin them.

## 2 Methods

### 2.1 Study Design and Data Collection

We undertook a descriptive study with a two‐stage sequential design, utilising online qualitative research methods (Germain et al. 2018) to allow us to capture and analyse online data from bottle brand websites. These methods included transparency concerning adopted search strategies, development of criteria for data selection and collection and assimilation of analysis between researchers. In essence, we ensured the rigour applied to our online research was akin to what would be applied to offline research.

#### 2.1.1 Stage One

We identified the 10 most popular bottles marketed in the United Kingdom for breastfeeding. We searched Google, Yahoo and Bing using Chrome on a cleared Internet browser (clear of cache, cookies and history) and the key words/phrases including: ‘best bottle for breastfeeding’ ‘breast and bottle feeding’, ‘mixed feeding’ and ‘combination feeding’. We recorded the top 30 bottle brand website hits for each keyword search (a total of 1350 website hits) on an Excel spreadsheet establishing popularity by number of hits. We searched across social media platforms Facebook, Tik Tok, Instagram, YouTube, X, online forums Reddit and Mumsnet.co.uk and Amazon.co.uk using the phrase ‘best bottle for breastfeeding’ to cross‐reference our findings. Bottle brands were excluded if they did not meet our inclusion criteria: needed to have a UK consumer website, consumers could access the website and information without registering, bottles could be purchased from UK retailers (supermarkets, department stores, online shops) or directly from the brand website, bottles were marketed for healthy, term infants.

We captured marketing materials pertaining to the bottles from the brand websites. One researcher searched text, visual and video content across tab headings including Bottle feeding, Breastfeeding, Parenting, Health professionals, Blogs, Reviews, and The Science. Data (marketing materials) were extracted by brand using copy/screen shots/snips and imported into NVivo11 (lumivero.com) for analysis. This exercise was repeated 3 months later by another researcher from the team. At this point it was found that Medela had removed all of their marketing materials pertaining to bottles and teats for healthy babies from their website. This was due to a review of their marketing guidelines to adhere to the WHO code. We excluded Medela from the study and included the next most popular brand.

We checked for assimilation of marketing materials between brand websites and UK retailers. All of the identified bottle brands were searched online to ‘buy’. Marketing materials used by UK retailers were usually in a condensed format compared to the brand webpages; however, consistency of information with brand websites was found to be 100%. This indicated that the brand websites were the ‘source’ of retail marketing materials and that consumers would be exposed to the same information.

#### 2.1.2 Stage 2

We captured the evidence that brand websites cited to underpin bottle features which claimed to align to breastfeeding and imported this into NVivo11.

### 2.2 Data Analysis

A research team member coded the data which described the bottle features which claimed to be aligned to breastfeeding. Overlapping codes were merged and seven main bottle features aligned to breastfeeding were established. This exercise was repeated by another researcher which resulted in adding the feature ‘Impacts positively on breastfeeding’ which resulted in eight main marketed bottle features claimed to be aligned to breastfeeding.

Next, we critically appraised the quality of the scientific evidence cited by the brands to support the marketed bottle features. A research team member reviewed potential sources of evidence including research publications, reports, surveys and professional reviews/commentaries that were specifically referenced from the brand websites, searching links, references, footnotes and relevant tabs. Evidence was excluded from further review if it did not meet our inclusion criteria: needed to be able to be discoverable by the general public, written in English and cited on the brand website to support their claims around bottle features aligned to breastfeeding. Critical Appraisal Checklists were used from the Johanna Briggs Institute (JBI) (JBI.global) for evidence synthesis to assess for methodological inclusion of studies. For those studies that met the JBI criteria, critical appraisal was conducted using the relevant JBI tools based on the type of evidence reported. Both researchers independently reviewed and extracted data on methodology and population type, the results and potential sources of bias. The quality of each source was assessed using items from the relevant methodological checklists. Given the purpose of the appraisal was not to include or exclude sources of evidence from a final synthesis based on their quality or risk of bias, an overall quality score was not assigned to sources. Instead, the authors discussed the level of quality of each source following independent review to draw conclusions about the overall quality of sources used by bottle brands.

### 2.3 Ethics Statement

The authors have nothing to report.

## 3 Results

### 3.1 Most Popular Bottle Brands

We identified 10 bottles: Tommee Tippee Closer to Nature/Natural Start, Minbie premium PPSU, Phillips Avent Natural Response, MAM Easy start Anti‐colic, Lansinoh Anti‐colic with Natural wave teat, Dr. Browns Options +, Nuby Combat Colic, NUK first choice, Emulait Classic/Anatomy and Nanobebe Breastmilk Flexy silicone. All could be purchased from the brand websites and across various UK retailers (supermarkets, chemists, online marketplaces) except for Minbie and Emulait, which could only be purchased from their brand websites.

### 3.2 Features of Bottles Associated With Breastfeeding

We established eight main features marketed as associated with breastfeeding: prevents/reduces nipple confusion, prevents/reduces bottle refusal, aids combi‐feeding, mimics breast/nipple, mimics physiology of breastfeeding, aids latch, high teat acceptance and positively impacts breastfeeding. Not every brand marketed every feature (see Table 1) with the most commonly marketed features being aids combi‐feeding, mimics breast/nipple and mimics physiology of breastfeeding. For the purpose of this study bottles are identified as follows: Tommee Tippee = TT, Minbie = MB, Phillips = PH, MAM = MM, Lansinoh = LS, Dr. Browns = DB, NUK = NK, Nuby = NU, Nanobebe = NB, Emulait = EM.

**Table 1:** Marketed bottle features related to breastfeeding by brand.

Id	Prevents/reduces nipple confusion	Prevents/reduces bottle refusal	Aids combi‐feeding	Mimics breast/nipple	Mimics physiology of breastfeeding	Aids latch	High teat acceptance	Has positive impact on breastfeeding
TT	√	√	√	√	√		√	
MB	√	√	√	√	√	√		√
PL			√	√	√		√	√
MM		√	√	√	√		√	
LS	√		√	√	√			√
DB	√		√	√	√	√	√	√
NY			√	√		√		
NK			√	√	√			
NB	√		√	√		√	√	
EM			√	√	√	√		

#### 3.2.1 Prevents or Avoids Nipple Teat Confusion

Half of the brands (*N* = 5) stated that their bottle either prevented or avoided nipple/teat confusion, with some brands claiming this was clinically proven,

Our breast‐like teats are specially designed to prevent nipple confusion (TT) Clinically proven to avoid nipple confusion in established breastfed babies (LS)

#### 3.2.2 Prevents or Solves Bottle Refusal, High Teat Acceptance

Half of the brands (*N* = 5) described their teats being associated with high levels of acceptance. This was designated an important feature of bottles in terms of managing bottle refusal by a breastfed baby,

95% of mums say their baby easily accepted our teat (TT)) 94% of babies accept the MAM teat with skinsoft surface (MM). After hearing from thousands of mums and seeing their tears of joy when Minbie solved their baby's bottle refusal (MB)

#### 3.2.3 Helps Combi‐Feed

All brands (*N* = 10) claimed that their bottle helped with ‘combi‐feeding’,

Clinically tested for an optimal combination of breast and bottle feeding (NK) The new First Flow 0 Month Teat and 2 oz/60 ml bottle, inspired by natural breast physiology, helps parents to combine breast‐ and bottle‐feeding (PH)

Brands also described their bottles as easing the ‘transition’ from breast to bottle, with bottle feeding being the natural progression and something to strive for,

Easy latch teats ease the transition from breast to bottle feeding (NY) The only bottle designed for breastmilk: baby instinctively connects to the breast shape, encourages smooth transition between breast and bottle (NB) I have been able to breast & bottle feed and I put that down to the Minbie teat (MB)

Information on the negative effect on breastfeeding of introducing partial bottle feeding as required by the WHO code and English regulations was not evident in any of the marketing data captured. In addition, the WHO Code requirement to include information on the benefits and superiority of breastfeeding was again not evident in the marketing materials captured.

#### 3.2.4 Mimics the Breast/Nipple

All of the brands (*N *= 10) made comparisons between their bottles and a human breast/nipple, describing equivalence between the two,

flexes and stretches like a breast (NY) unique soft‐nipple texture, resembling the breast and human skin (PS) Flesh like in both softness and texture, the nipple stretches and elongates during bottle feeding feeding…Our nipples [teats] replicate the texture of simulated areola glands and have interior fibres to mimic milk ducts (EM)

These comparisons were augmented by descriptions of the bottles providing the baby with a physical experience equivalent to breastfeeding,

[teat] modelled on a mother's nipple as she breastfeeds. Giving tongue and jaw enough room ‐ and the feeling babies love (NK) …this squeezable bottle feels just like mum and provides baby with the familiar warmth and comfort of mums breast (TT)

One bottle brand (EM) advertised a 3D scanning service for mothers to enable them to purchase the teat closest to their own nipple, with five skin tones available,

The scan utilizes unique 3D algorithms to capture key nipple measurements contours and colours to tailor your anatomy for a bottle that is yours, truly (EM)

#### 3.2.5 Mimics the Physiology of Breastfeeding

Virtually all of the brands (*N* = 8) described their bottles exhibiting features of the physiology of breastfeeding. Some of these were centred around the teats allowing the baby to control the feed,

The special NUK First Choice+ Flow Control teat lets babies control the flow themselves when they are drinking (NK) [teat] …delivers a more controlled flow rate closer to the breast‐flow average (PS)

Others focused on babies being naturally ‘programmed’ to breastfeed and that their teats could match this,

The complexity of newborn feeding demands a bottle teat that perfectly matches the way newborns want and need to feed on the breast. Our unique teat design is the only one that achieves this (MB) The natural wave teat enables the baby to maintain the natural sucking styles learnt at the breast (LN)

Only two brands referenced ‘responsive feeding’ in the marketing materials of their bottles. Both gave no further information on the principles of responsive bottle feeding, with one of the brands (EM) depicting a visual of a baby being fed lying flat on its back and another with a mother feeding their baby from behind, removing eye contact, both of which are inconsistent with responsive bottle feeding guidance.

#### 3.2.6 Aids Latch, Positively Impacts Breastfeeding

Half of the brands (*N* = 5) marketed their teats as encouraging ‘latch,’ a term usually associated with breastfeeding,

Our bottles have a breast‐like feel that encourages a natural latch (TT) Contoured breast‐like teat for a proper latch and more natural feeding experience (DB)

A number of the brands (*N* = 4) described their bottles as having a positive impact on breastfeeding,

Scientifically proven: the Natural Wave Teat helps the baby maintain the natural sucking style learnt at the breast …and preserve established breastfeeding patterns (LN) Strengthens your babies breastfeeding co‐ordination…. Enhances and protects your baby's mother baby breastfeeding (MB) Encourages baby to still want to feed from the breast (PL)

### 3.3 Evidence to Support Marketed Features of Bottles

Manufacturer evidence used to support their bottle feature claims included parent and health professional testimonials, market research, blogs and reviews. Of the 10 bottle brands we identified, only 3 (NUK = 4, Dr Browns = 2 and Lansinoh = 1) cited scientific research evidence to support the claims made about their bottles. The other seven companies cited no scientific evidence for any of the claims they made (see Table 2). Of the three brands citing scientific supporting evidence only seven scientific sources were used to support their claims. The methodological quality of the included sources (quasi‐experimental studies *(n* = 3), qualitative studies (*n* = 1), prevalence studies (*n* = 2) and textual evidence (*n* = 1) was assessed based on JBI critical appraisal tools. The most common claim to be supported by scientific evidence was that bottle feeding replicates the physiology of breastfeeding. The methodological quality of the seven research evidence types cited by companies ranged from low to moderate with only one deemed to be of high quality. In the majority of empirical studies, there was a lack of methodological detail reported, leading the authors to question the risk of bias in those studies.

**Table 2:** Scientific evidence to support bottle features by brand.

Id	Prevents/reduces nipple confusion	Prevents/reduces bottle refusal	Aids combi‐feeding	Mimics breast/nipple	Mimics physiology of breastfeeding	Aids latch	High teat acceptance	Positive impact on breastfeeding
TT	X	x	x	x	x	N/A	x	N/A
MB	X	x	x	x	x	x	N/A	x
Ph	N/A	N/A	x	x	x	N/A	x	x
MM	N/A	x	x	x	x	N/A	x	N/A
LS	x	x	x	x	Woolridge (2011)	N/A	N/A	x
DB	X	N/A	Francis et al. (2008)	x	x	x	Rose (n.d.)	x
NY	N/A	N/A	x	x	N/A	x	N/A	N/A
NK	N/A	N/A	x	Jütte et al. (2014)	Moral et al. (2010) Arsenina et al. (2006) Herrmann (2010)	N/A	N/A	N/A
NB	x	N/A	x	x	N/A	x	x	N/A
EM	N/A	N/A	x	x	x	N/A	N/A	N/A

Abbreviations: N/A = not applicable as brand did not market this feature, X = no evidence found.

We undertook further analysis to investigate if the studies included on the brand sites assimilated with the bottle features they were cited to support. Four of the seven studies were not of direct relevance. The aim of Moral et al. (2010) study was to show different sucking patterns in bottle fed, breastfed and mixed fed babies rather than how well the NUK teat mimicked the physiology of breastfeeding. Although similarities were found between breastfeeding and bottle feeding using the Lansinoh teat in Woolridge (2011) study, the authors stated that their ‘overriding impression was that babies showed individually‐characteristic styles of feeding that were not wholly explicable in terms of either the teat characteristics, or the rate of milk flow’. Jütte et al. (2014) study focused on the physiology of milk duct orifices and Rose (n.d.) compared bottles with bottles and did not compare them with breastfeeding.

## 4 Discussion

Our study has investigated features of infant feeding bottles marketed in the United Kingdom to replicate breastfeeding and the evidence that underpins them. It presents a troubling picture, with claims that bottles have equivalent features to breastfeeding and almost non‐existent scientific evidence to support these features. When scientific evidence was found, it was generally outdated, exhibited small sample sizes and lacked methodological detail, incurring risk of bias. In addition, some of the evidence did not support the bottle feature it was claimed to be aligned to. This does not appear to fully comply with the Advertising and Standards Agency Nonbroadcast Code on misleading advertising, a set of rules that when advertising online, for example, businesses must comply with regarding accuracy and honesty of their marketing materials (ASA.org). More specifically, rule 3.7 on ‘Ensuring the substantiation of objective claims’, which states that businesses should ensure that they hold evidence to support the claims that consumers are likely to regard as objective.

The lack of scientific research cited by the bottle companies reflects the general status of evidence related to many of the features that were being marketed. Studies pertaining to nipple confusion continue to be limited and inconclusive (Zimmerman and Thompson 2015). This is exacerbated by the existence of nipple confusion remaining open to debate by breastfeeding mothers themselves (redacted). However, bottle companies continue to exploit parental concerns around the impact of nipple confusion on breastfeeding, designating their bottles as preventing or avoiding it.

Only two studies having been published on bottle refusal by breastfed babies. One study found limited success when using different bottles and teats as a method to try to solve bottle refusal < 15% (redacted). The other study pointed to multi‐factorial reasons being behind refusal, such as babies making a preference for the breast and their caregiver, rather than it being based on rejection or preference of a bottle brand (redacted). Bottle companies have used the ‘pathologising’ of bottle refusal by breastfed babies to their advantage, and as seen in our study, make unfounded claims around their bottles being able to solve it, citing ‘high acceptance’.

The design and marketing of bottles that facilitate combi‐feeding through replication of the physiology of breastfeeding was a feature across all bottle brands. However, there remains a lack of consensus between researchers and experts on how babies feed at the breast, thus designing the optimum bottle to combine seamlessly with breastfeeding is problematic. Once viewed as two very distinct mechanisms of feeding, more recent studies describe similarities between bottle and breast feeding (Kotowski et al. 2020). Of interest is Woolridge's suggestion that although some babies do adopt similar feeding patterns between breast and bottle, feeding style is most likely individual to babies (Woolridge 2011). This is predictably unaccounted for in any of the marketing information we reviewed.

Our study found a universal emphasis on bottles being marketed to physically replicate a breast, with skin tones and AI bespoke generated teats being marketed. Few studies have compared the physical properties of breast to bottle. Original work by Nowak et al. highlighted the inability of a bottle teat to elongate like a human nipple, although this is now claimed to be a feature of the Emulait bottle albeit with no identifiable underpinning research. Other more obvious differences come in the guise of the unique temperature, smell, shape and ‘agility’ of a breast that continue to evade bottle design.

Our study highlights many areas where marketing of bottles and teats has contravened the WHO code (1981) and appears not to adhere to UK regulations (ASA.org). This was evident in the marketing and idealisation of the bottles and misleading equivalence to breastfeeding, and in the omission of information around the impact partial bottle feeding may have upon breastfeeding. These breaches echo findings from Alcaire et al. (2021) in their study of the labelling and marketing of bottles and teats in Uruguay, and recently Theurich, Ziebart, and Strobl et al. (2024) in their cross‐sectional survey of infant feeding bottles in Germany. They have also been highlighted previously by Baby Milk Action (babymilkaction.org) and IBFAN (ibfan.org). However, it is unsurprising that these violations occur, given the current weak UK regulation around the marketing of bottles and lack of emphasis on bottles in the WHO code. In addition, regulators have not kept pace with bottle manufacturer developments.

Stronger legislative controls by the UK Government surrounding the marketing of bottles to protect breastfeeding are urgently needed and there should be a particular focus on digital information and advertising, which parents' often utilise rather than advice from health professionals (Kubb and Foran et al. 2020). This is important due to the implications of caregivers making decisions around bottle feeding their baby based off manufacturer's claims. This in turn poses public health implications due to babies not being breastfed and potentially impacts breastfeeding initiation, duration and cessation. Information provided by bottle companies to purchasers needs to be transparent in terms of scientific underpinning evidence to enable decision making around infant feeding to be fully informed.

The are limitations to our study. Due to the transient nature of online marketing, website information is constantly changing. Our study relates to the most popular bottles marketed in the United Kingdom which may impede transfer of results; however, the limited body of work around this subject undertaken outside of the United Kingdom concurs with many of our findings, therefore strengthening our methodological choices.

## 5 Conclusion

Infant feeding bottles are being marketed as ‘equivalent’ to breastfeeding; however, our study shows that the scientific evidence used to support claims of ‘breast‐like’ features of these bottles is almost non‐existent, misleading and inadequate. Greater awareness of the lack of evidence to support advertised features of bottles that are claimed to be aligned to breastfeeding is required, so that those who plan to, or who are breastfeeding, can make informed decisions around their use. In line with this, research is needed on the impact of the marketing of bottles on decisions and practices around breastfeeding and more effective regulation of bottle company advertising is urgently required.

## Author Contributions

C.M., B.S. and K.B. contributed to conceptualisation. C.M. undertook funding acquisition and designed the methodology. C.M., B.S. and K.B. undertook the investigation and performed formal analysis. C.M. led the writing of the manuscript, B.S. and K.B. contributed to reviewing and editing. All authors have read and approved the final manuscript.

## Conflicts of Interest

The authors declare no conflicts of interest.

## Data Availability

Researchers wishing to undertake additional analyses of the data are invited to contact the corresponding author.

## Endnotes

1 We define combi‐feeding as feeding by breast and bottle alongside each other. The bottle may contain expressed breastmilk or formula or both.

2 We define breastmilk feeding as the feeding of expressed breastmilk not at the breast.

## References

[mcn70008-bib-0001] asa.org . “Advertising Standards Agency Non‐Broadcast Code Available.” Accessed August 27, 2024. https://www.asa.org.uk/type/non_broadcast/code_section/03.html.

[mcn70008-bib-0002] Alcaire, F. , L. Antúnez , and L. Vidal , et al. 2021. “The Idealisation of Bottle Feeding: Content Analysis of Feeding Bottles and Teats Packages in Uruguay.” Public Health Nutrition 24, no. 10 (July): 3147–3155. 10.1111/mcn.13632. 33087203 PMC9884790

[mcn70008-bib-0003] Allen, E. , A. R. Rumbold , A. Keir , C. T. Collins , J. Gillis , and H. Suganuma . 2021. “Avoidance of Bottles During the Establishment of Breastfeeds in Preterm Infants.” Cochrane Database of Systematic Reviews 2021, no. 10 (October): 005252. 10.1002/14651858.CD005252.pub5. PMC8529385 34671969

[mcn70008-bib-0032] Arsenina, O. J. , A. G. Nadtochiy , and N. V. Popova . 2006. Study of the Act of Sucking in Healthy Babies in Breastfeeding and Artificial Feeding Using NUK Teats. 14–16. https://nuk.de/fileadmin/local/DE/about_NUK/research/pdf/Ortodontia_engllische_Version_26112009_final_.pdf.

[mcn70008-bib-0004] babymilkaction.org . 2017. “Baby Milk Action, IBFAN UK Look What They're Doing. How Marketing of Feeding Products for Infants and Young Children in the UK Breaks the Rules.” Accessed August 26, 2024. https://www.babymilkaction.org/archives/12873.

[mcn70008-bib-0005] Forster, D. A. , H. M. Johns , H. L. McLachlan , A. M. Moorhead , K. M. McEgan , and L. H. Amir . 2015. “Feeding Infants Directly at the Breast During the Postpartum Hospital Stay Is Associated With Increased Breastfeeding at 6 Months Postpartum: A Prospective Cohort Study.” BMJ Open 5, no. 5 (May): e007512. 10.1136/bmjopen-2014-007512. PMC4431142 25953728

[mcn70008-bib-0033] Francis, J. , K. Rogers , P. Brewer , D. Dickton , and R. Pardini . 2008. “Comparitive Analysis of Ascorbic Acid in Human Milk and Infant Formula using varied milk delivery systems.” International Breastfeeding Journal 3: 1–6. 10.1186/1746-4358-3-19. 18694505 PMC2526073

[mcn70008-bib-0006] Germain, J. , J. Harris , S. Mackay , and C. Maxwell . 2018. “Why Should We Use Online Research Methods? Four Doctoral Health Student Perspectives.” Qualitative Health Research 28, no. 10: 1650–1657. 10.1177/1049732317721698. 28745106

[mcn70008-bib-0007] Gov.UK . 2016. “Commission Delegated Regulation (EU) 2016/127 Article 11 Requirements on Information Relating to Infant and Young Child Feeding.” Accessed August 26, 2024. https://www.legislation.gov.uk/eur/2016/127/article/11.

[mcn70008-bib-0034] Herrmann, C. 2010. “Oethodontic teats. A study overview‐background, data, facts and results for the NUK system.” 1–3. https://www.nuk.co.uk/fileadmin/Default/about_NUK/research/pdf/PR-Herrmann_final_GB_March2010.pdf.

[mcn70008-bib-0009] JBI.global . “JBI Critical Appraisal Tools.” https://jbi.global/critical-appraisal-tools.

[mcn70008-bib-0010] Jütte, J. , A. Hohoff , C. Sauerland , D. Wiechmann , and T. Stamm . 2014. “In Vivo Assessment of Number of Milk Duct Orifices in Lactating Women and Association With Parameters in the Mother and the Infant.” BMC Pregnancy and Childbirth 14: 124. 10.1186/1471-2393-14-124. 24694236 PMC3992155

[mcn70008-bib-0011] Kotowski, J. , C. Fowler , C. Hourigan , and F. Orr . 2020. “Bottle‐Feeding an Infant Feeding Modality: An Integrative Literature Review.” Maternal & Child Nutrition 16, no. 2: e12939. 10.1111/mcn.12939. 31908144 PMC7083444

[mcn70008-bib-0012] Kubb, C. , and H. M. Foran . 2020. “Online Health Information Seeking by Parents for Their Children: Systematic Review and Agenda for Further Research.” Journal of Medical Internet Research 22, no. 8: e19985. https://pmc.ncbi.nlm.nih.gov/articles/PMC7479585/. 32840484 10.2196/19985 PMC7479585

[mcn70008-bib-0013] lumivero.com . “NVivo11.” https://lumivero.com/products/nvivo/.

[mcn70008-bib-0014] McAndrew, F. , J. Thompson , L. Fellows , M. Speed , and M. Renfrew . 2012. “Infant Feeding Survey 2010.” Accessed May 15, 2024. http://www.hscic.gov.uk.

[mcn70008-bib-0015] McCoy, M. B. , and P. Heggie . 2020. “In‐Hospital Formula Feeding and Breastfeeding Duration.” Pediatrics 146, no. 1: e20192946. 10.1542/peds.2019-2946. 32518168

[mcn70008-bib-0016] Michalopoulou, S. , A. L. Garcia , L. Wolfson , and C. M. Wright . 2024. “Does Planning to Mixed Feed Undermine Breastfeeding?” Maternal & Child Nutrition 20, no. 2 (April): e13610. 10.1111/mcn.13610. 38093405 PMC10981487

[mcn70008-bib-0017] Moral, A. , I. Bolibar , G. Seguranyes , et al. 2010. “Mechanics of Sucking: Comparison Between Bottle Feeding and Breastfeeding.” BMC Pediatrics 10, no. 6: 6. http://www.biomedcentral.com/1471-2431/10/6. 20149217 10.1186/1471-2431-10-6 PMC2837866

[mcn70008-bib-0018] Neifert, M. , R. Lawrence , and J. Seacat . 1995. “Nipple Confusion: Toward a Formal Definition.” Journal of Pediatrics 126: S125–S129. https://pubmed.ncbi.nlm.nih.gov/7776072/. 7776072 10.1016/s0022-3476(95)90252-x

[mcn70008-bib-0020] Pérez‐Escamilla, R. , A. Hromi‐Fiedler , and E. C. Rhodes , et al. 2022. “Impact of Prelacteal Feeds and Neonatal Introduction of Breast Milk Substitutes on Breastfeeding Outcomes: A Systematic Review and Meta‐Analysis.” Supplement, Maternal & Child Nutrition 18, no. S3 (May): e13368. 10.1111/mcn.13610. 35489107 PMC9113480

[mcn70008-bib-0021] Rollins, N. , E. Piwoz , and P. Baker , et al. 2023. “Marketing of Commercial Milk Formula: A System to Capture Parents, Communities, Science, and Policy.” Lancet 401 (February): 486–502. 10.1016/S0140-6736(22)01931-6. 36764314

[mcn70008-bib-0022] Rose, M. n.d. “Clinical Research Trial of Bottle Nipples: Choosing the Best Nipple for the Individual Neonate.” Accessed May 5, 2024. https://www.drbrownsmedical.com/wp-content/uploads/2016/08/dbm-choosing-the-best-nipple-article-proof_1.pdf.

[mcn70008-bib-0023] Theurich, M. A. , M. Ziebart , and F. Strobl . 2024. “National Survey of Infant Feeding Bottles in Germany: Their Characteristics and Marketing Claims.” Maternal & Child Nutrition 20, no. 3 (July): e13632. 10.1111/mcn.13632. 38385989 PMC11168357

[mcn70008-bib-0024] Ventura, A. K. , R. Li , and X. Xu . 2020. “Associations Between Bottle‐Feeding During Infancy and Obesity at Age 6 Years Are Mediated by Greater Infancy Weight Gain.” Childhood Obesity 16, no. 5 (July): 316–326. https://www.ncbi.nlm.nih.gov/pmc/articles/doi/10.1089/chi.2019.0299. 32498550 10.1089/chi.2019.0299

[mcn70008-bib-0025] Victora, C. G. , R. Bahl , A. J. D. Barros , et al. 2016. “Breastfeeding in the 21st Century: Epidemiology, Mechanisms, and Lifelong Effect.” Lancet 387: 475–490. 10.1016/S0140-6736(15)01024-7. 26869575

[mcn70008-bib-0026] WHO . 1981. “International Code of Marketing of Breastmilk Substitutes.” Accessed May 12, 2024. https://iris.who.int/bitstream/handle/10665/254911/WHO-NMH-NHD-17.1-eng.pdf.

[mcn70008-bib-0027] WHO . 2023. “Infant and Young Child Feeding.” Accessed December 10, 2024. https://www.who.int/news-room/fact-sheets/detail/infant-and-young-child-feeding.

[mcn70008-bib-0028] WHO, UNICEF, IBFAN . 2024. “Marketing of Breast‐Milk Substitutes National Implementation of the International Code, Status Report 2024.” Accessed December 10, 2024. https://iris.who.int/bitstream/handle/10665/376854/9789240094482-eng.pdf?sequence=1.

[mcn70008-bib-0029] Woolridge, M. 2011. “Leeds Ultrasound Imaging Study 2 (LUIS2): How Breasted Babies Bottle Feed.” School of Healthcare, University of Leeds. Accessed July 7, 2023. https://lansinoh.sk/wp-content/uploads/2017/11/WoolridgeLansinoh_Final_Report_Body_v1_32.pdf.

[mcn70008-bib-0030] Zheng, M. , A. J. Cameron , C. S. Birken , et al. 2020. “Early Infant Feeding and BMI Trajectories in the First 5 Years of Life.” Obesity 28: 339–346. https://onlinelibrary.wiley.com/doi/epdf/10.1002/oby.22688. 31970916 10.1002/oby.22688

[mcn70008-bib-0031] Zimmerman, E. , and K. Thompson . 2015. “Clarifying Nipple Confusion.” Journal of Perinatology 35, no. 11: 895–899. 10.1038/jp.2015.83. 26181720

